# Influence of maternal and paternal pre-conception overweight/obesity on offspring outcomes and strategies for prevention

**DOI:** 10.1038/s41430-021-00920-7

**Published:** 2021-06-15

**Authors:** Bettina Hieronimus, Regina Ensenauer

**Affiliations:** 1 Institute of Physiology and Biochemistry of Nutrition, Max Rubner-Institut, Federal Research Institute of Nutrition and Food, Karlsruhe, Germany; 2 Institute of Child Nutrition, Max Rubner-Institut, Federal Research Institute of Nutrition and Food, Karlsruhe, Germany

**Keywords:** Risk factors, Metabolic disorders

## Abstract

Overweight, obesity, and their comorbidities remain global health challenges. When established early in life, overweight is often sustained into adulthood and contributes to the early onset of non-communicable diseases. Parental pre-conception overweight and obesity is a risk factor for overweight and obesity in childhood and beyond. This increased risk likely is based on an interplay of genetic alterations and environmental exposures already at the beginning of life, although mechanisms are still poorly defined. In this narrative review, potential routes of transmission of pre-conceptional overweight/obesity from mothers and fathers to their offspring as well as prevention strategies are discussed. Observational evidence suggests that metabolic changes due to parental overweight/obesity affect epigenetic markers in oocytes and sperms alike and may influence epigenetic programming and reprogramming processes during embryogenesis. While weight reduction in overweight/obese men and women, who plan to become pregnant, seems advisable to improve undesirable outcomes in offspring, caution might be warranted. Limited evidence suggests that weight loss in men and women in close proximity to conception might increase undesirable offspring outcomes at birth due to nutritional deficits and/or metabolic disturbances in the parent also affecting gamete quality. A change in the dietary pattern might be more advisable. The data reviewed here suggest that pre-conception intervention strategies should shift from women to couples, and future studies should address possible interactions between maternal and paternal contribution to longitudinal childhood outcomes. Randomized controlled trials focusing on effects of pre-conceptional diet quality on long-term offspring health are warranted.

## Introduction

The health of a child depends on its environment. Exposure to suboptimal conditions early in life appears to affect the health not only momentarily but throughout a life course. The earliest environment is defined by the mother and, amongst other factors, both under- and overnutrition during pregnancy can lead to undesirable health outcomes in offspring. The observation that early-life exposure during pregnancy and infancy impacts life-long health led to the generation of the Developmental Origins of Health and Disease hypothesis [1]. This theory is widely accepted today and resulted in a boost in research focusing on pregnant women. However, recent evidence points to the pre-conception and/or peri-conception phase as an important time to influence offspring health outcomes. The peri-conceptional phase encompasses the time that precedes, includes, and immediately follows conception [2].

With ever-increasing numbers, overweight and obesity in children and their comorbidities have become a major threat to personal and societal wellbeing in high-, middle- and low-income countries alike [3, 4]. Parental peri-conception overweight is a strong predictor for increased birth weight as well as for childhood overweight and obesity [5–11]. While the causes for obesity are multifactorial and include effects of (food) environment, socioeconomic status, psychological health, and cultural aspects [12], results from adoption studies suggest that overweight and obesity also have a genetic component [13]. Mechanisms underlying overweight development and fetal adipogenic programming through influences of early-life stages are still poorly understood.

In this review, we focus on parental overweight/obesity in the peri-conceptional phase and its effects on gamete quality and epigenetic changes as mediators of increased risk for overweight or obesity in offspring. We further reviewed risks associated with pre-conceptional weight loss in overweight or obese mothers and fathers and discuss improvement of the diet as a possible alternative to body mass index (BMI) reduction.

## Methods: sources and selection criteria

The articles selected for this review were English-language, full-text articles and abstracts that were identified by a series of PubMed and EMBASE searches using keywords either alone or in combination and published before July 31, 2020. The keywords used included *obesity, overweight, child outcomes, infant outcomes, large for gestational age, small for gestational age (SGA), pregnancy, pre-conception, weight loss, bariatric surgery, micronutrient, oocyte quality, sperm quality, seminal fluid, assisted reproductive technologies, epigenetics, epigenetic programming, DNA methylation*, and *gestational weight gain*. The reference lists of articles and reviews identified by this search strategy were also searched, and those articles that deemed to be relevant were selected. This review is not exhaustive; therefore, we provide references for more in-depth reviews when we thought it might be helpful or appropriate. We used the words ‘mother’ and ‘father’ as an indication of the biological sex of the respective parent and not as a representation of their social gender.

## Why is pre-conception health important?

The decision to have a child is life changing. However, about 40% of pregnancies are unintended [14], and these pregnancies seem to be associated with a higher risk for undesirable pregnancy and birth outcomes [15]. A recent systematic review reported that during the pre-conception phase and pregnancy, women may not meet recommendations for vegetable, cereal grain, vitamin, and mineral intake [16]. Even amongst those who plan to get pregnant, awareness of pre-conception health and motivation for behavioral changes appears limited in both women and men [16–18]. This evidence suggests that many women and men might not have optimal metabolic conditions prior to pregnancy. Overweight and obesity have become major global problems [3, 19]. Among women of childbearing age, up to 70% are overweight or obese and up to 40% are obese worldwide [19], and these numbers are predicted to increase [20, 21]. Therefore, pregnant women with overweight or obesity are not a minor instance but have become part of a new norm [19].

Obesity is a risk factor for a wide range of complications related to pregnancy. First, it causes in- and subfertility in men and women [22, 23]. In addition, pregnant women who are obese have higher rates of hypertension, preeclampsia, thromboembolism, cesarean section, and excessive weight gain during pregnancy and have increased rates of metabolic abnormalities such as gestational diabetes (reviewed in [24, 25]). Their children are more at-risk to be born large for gestational age or SGA, to have congenital defects, or to develop overweight and/or cardiometabolic diseases later in life (reviewed in [6, 7, 25, 26]). Studies that assessed the effect of the mothers’ high pre- or early-pregnancy BMI show a positive association with increases in birth weight, longitudinal BMI development during preschool age [27–29], and body weight in offspring up to age 18 years [7, 10]. In addition, evidence is emerging suggesting that paternal BMI may affect offspring risk for obesity or type 2 diabetes across the life course [5, 30]. However, high-quality studies are sparse.

Overweight and obesity developed in childhood and adolescence are generally sustained in adulthood and, if established already early in life, often progress to more severe stages over the course of a lifetime [31]. Overweight and obesity seem to be transferred from ancestor generations to their progeny, with data indicating that a susceptibility for overweight can be passed on over three generations, through environmental exposures modulating the germline epigenome and leading to a transgenerational phenotype [32]. Here, we focus on parent-to-child transmission. To overcome the associated personal and societal costs (psychological and financial), the intergenerational cycle has to be broken to stop the alarming rate of global obesity development. Lifestyle intervention studies in women with overweight and obesity during their pregnancy failed to prevent undesirable offspring outcomes at birth [33, 34]. Therefore, weight should be reduced prior to pregnancy, as very early embryonic development, when women might not yet know that they are pregnant, seems to be essential for pregnancy success and life-long health of a child [34]. The low adherence to pre-pregnancy health guidelines [16] and the high proportion of unplanned pregnancies [14] further strengthen the urgency to implement strategies for improvement of pre-conception health.

## Gamete quality

The life of all vertebrates starts with the fusion of ovum and sperm. The gametes’ quality and potential are inherently affected by the health status of the individual who generates them. While women often are in the spotlight when it comes to the health of a growing life, the male counterparts contribute equally to the genetic material. Overweight and obesity affect the quality of oocytes and sperms alike (see below). Several studies demonstrated epigenetic changes in DNA and histone methylation, histone acetylation, and microRNA expression in oocytes and sperms. Epigenetic modifications change the way genes are expressed without altering the genetic code itself. In changing environments, these potentially reversible modifications might be beneficial over genetic alterations [35]. They are believed to be an adaptation mechanism that provides an evolutionary advantage for the subsequent generations.

## Oocyte

The maturation process from primordial follicles to ovulation takes several months and is regulated by hormonal signals. The complete process remains to be fully understood. During the months of maturation, the oocyte and its surrounding cumulus cells are exposed to the nutritional and hormonal environment within the woman’s body [36]. Overweight and obesity affect sex hormones, oogenesis and fertility, and, within an oocyte, gene expression, epigenetic markers, and mitochondria [37–39].

The unique contribution of the oocyte quality to offspring health as opposed to the maternal metabolic environment during early-life development can hardly be assessed in natural pregnancies. However, egg retrievals are routinely performed in women undergoing assisted reproductive technology (ART), and several studies assessed offspring outcomes after ART [40, 41]. Decreased pregnancy and live birth rates resulted from increasing autologous [42] as well as a donor [43] oocyte BMI, possibly pointing to a BMI-related impairment of the oocyte quality prior to fertilization.

While observational evidence on transmission of maternal obesity to an offspring is solid, data on mechanisms are sparse. A recent report focused on STELLA, a protein highly expressed in primordial germ cells [44]. High-fat diet (HFD)-induced obesity led to a reduction in STELLA in murine oocytes and a subsequent change in DNA methylation. Exogeneous overexpression of STELLA partly reversed methylation patterns as well as phenotypical obesity-associated defects during embryogenesis [44]. The insufficient amount of STELLA in oocytes from obese mice was suggested to be one route potentially contributing to the intergenerational transmission of maternal obesity to their offspring.

In addition, obesity-related overnutrition and alterations of metabolites (glucose, free fatty acids) in the circulation are assumed to be mirrored in the intra-follicular fluid with increased concentrations of lactate, insulin, leptin, and triglycerides [37, 45]. High intra-follicular insulin concentrations were related to adiposity in women and might potentially impair oocyte function [37, 46, 47]. Furthermore, within oocytes of obese mice and women, high intra-follicular lipid concentrations cause lipotoxicity [48, 49], and lipid accumulation was associated with impaired mitochondrial function and oxidative stress resulting in DNA damage [23, 38, 49]. In their recent review, Bradly and Swann concluded that a balance of fatty acid and pyruvate oxidation is a prerequisite for optimal oocyte function and early embryonic development, and a disruption of the balance—by excess or shortage of either substrate—will result in compromised oocyte and embryo quality [50]. However, not only the amount of lipids but also lipid quality could affect oocytes and pregnancy success. Higher palmitic acid and total saturated fatty acid concentrations were found in the intra-follicular fluid from degenerate bovine oocytes, whereas competent oocytes showed higher concentrations of linolenic acid [51]. Non-esterified fatty acids were higher in oocytes from obese than overweight and normal weight women, possibly affecting oocyte quality [52]. These results suggest that an adjustment of the nutritional quality of the oocyte environment might be able to dampen negative effects resulting from obesity.

## Sperm

Most interventions to improve pregnancy and child health target mothers, as their behavior from conception to the end of breastfeeding and onward affects the metabolic outcomes of their offspring. However, paternal pre-conception lifestyle seems to affect child outcomes as well. Children from pre-conceptionally obese fathers are likely to develop obesity and/or metabolic dysfunction independent of the mother’s weight [5, 30]. In mice, female offspring from pre-conceptionally obese fathers showed impaired insulin secretion and glucose tolerance [53]. Furthermore, paternal obesity was negatively associated with live birth rates in couples undergoing ART [54] and positively associated with changes in sperm, including alterations in small RNA species [55, 56].

Sperm carry non-coding RNAs, which might also modulate DNA methylation [57–59]. Obese male mice exhibited altered microRNA and DNA methylation profiles in sperm and transmission of adverse metabolic phenotypes, which were partly transferred to the following two generations [60]. A pre-conceptional weight loss study in obese male mice showed that changes in microRNA patterns in sperm are reversible through lifestyle changes (diet and/or exercise) and that these findings were associated with a decrease in the predisposition for adiposity and insulin resistance in female offspring [61]. Interestingly, in the Project Viva study, paternal BMI at conception was associated with methylation patterns in cord blood samples and in offspring blood at ages 3 and 7 years [9].

DNA methylation patterns and non-coding RNA content of sperm from obese men differed from those of lean men [62]. In the same report, sperm from obese men who underwent gastric bypass surgery showed DNA methylation changes in 1.509 genes 1 week after surgery (median BMI: 40.1 kg/m^2^, *p* < 0.05 compared with pre-surgery) in comparison to sperm obtained from the same individual 1 week before surgery (median BMI: 42.6 kg/m^2^) [62]. Of those, differences in 1.004 gene methylations were still evident 1 year after surgery when weight stabilized (median BMI: 33.9 kg/m^2^, *p* < 0.05 compared with pre-surgery) [62]. These findings suggest that epigenetic changes occur rapidly in sperm and that they are affected by body weight. Recently, it was discovered that seminal fluid has its own microbiome, which is affected by the diet [63, 64]. The microbiome composition may potentially also affect epigenetic markers in the sperm. However, the role of the seminal fluid microbiome remains to be elucidated.

Observational studies suggest that children born to two parents with overweight or obesity have a higher risk to develop obesity than children with only one overweight or obese parent [8, 65]. However, offspring’s weight at birth and at 3 years of age was stronger associated with maternal obesity than paternal obesity [66]. Future research needs to clarify whether good metabolic health of one parent could dampen adverse effects of an overweight/obese partner on the long-term outcome of the child.

## Epigenetic programming

Once sperm and oocyte merged, their DNA undergoes substantial epigenetic reprogramming. During the first week after fertilization, parental DNA methylation is widely removed from all cells, and de novo methylation occurs. These new epigenetic markers—i.e., epigenetic programming—are partly sustained over the course of a life and can affect metabolism [67]. Primordial germ cells, the cells that will become the gametes, undergo a second round of demethylation during embryogenesis [68].

Environmental exposures affect the process of epigenetic programming, which is apparent e.g. in children conceived through ART. Changes in epigenetic programming and undesirable pregnancy and health outcomes, including low birth weight, preterm birth, or birth defects, are increased after ART compared with spontaneous conception [69]. Limited evidence suggests long-term effects in offspring conceived through ART such as increased risks for cardiovascular diseases or decreased reproductive function [69]. One study found that children aged 0–4 years conceived via ART were smaller compared with those conceived naturally [40]. These differences were not detectable at 5 years of age or older [40], which indirectly suggests that the children conceived through ART might possibly develop accelerated growth in their first years of life. In ART, the environment for the zygote and during the first cell divisions is very different from the natural surroundings in a mother’s body. The culture medium itself affects offspring birth weight and weight trajectory in the first 2 years of life, as shown in a study where two different culture media for in vitro fertilization were tested [70, 71]. These results demonstrate that the intrauterine environment might be one route of intergenerational transmission of overweight or obesity through changes in epigenetic programming.

## Risks associated with weight reduction prior to conception

Considering the outlined risks associated with overweight and obesity, health guidelines recommend a normalization of the body weight prior to pregnancy [72, 73]. The reasonable amount of weight loss and safe strategies to achieve a reduction in BMI differ considerably, as recently reviewed [73]. Studies focusing on fertility showed improved fertility rates after modest weight loss of a mean of 6.6 ± 4.6 kg (mean 6.9% of initial body weight) [74], and higher losses (>10% of body weight) might benefit fertility further [75]. A study in mice assessed the effects of pre-conceptional weight loss on epigenetic modulators in placenta and fetal liver at embryonic day (E) 18.5 [76]. Prior to mating, a normal weight group was fed with a control diet for 4 months and compared with an overweight group, fed a HFD for 4 months, and a weight loss group, fed a HFD for 2 months followed by a control diet for 2 months. The mice were sacrificed at E18.5, and expression levels of epigenetic modulators in fetal liver, placental labyrinth, and uterine junctional zone were assessed. Long-term HFD led to a reduction in fetal weight, which could be partially rescued by diet-induced weight loss prior to mating. Weight loss further induced a partial normalization of expression levels of a subset of genes belonging to the epigenetic machinery [76]. This study suggests that epigenetic processes could be modulated by weight change prior to pregnancy, at least in mice. As this study ended during pregnancy, long-term effects on the offspring would be of interest.

In a rat model with a similar intervention, male offspring were followed up until 150 days of age [77]. Pubs from obese dams who were subjected to a dietary intervention prior to pregnancy showed metabolic parameters similar to pubs from dams in the control diet group at postnatal day 21. Later at day 150, their serum leptin levels as well as fat tissue weight and fat cell size were lowered compared to offspring of obese dams, although fasting insulin (day 120), body weight, and food intake (day 150) were not [77]. The weight of the dams in the dietary intervention group was not stabilized, which might have affected offspring outcomes.

Clinical trials assessed the effects of severe weight loss after bariatric surgery on pregnancy and child outcomes. These results raise a note of caution for pre-conceptional weight loss. In addition to higher risks of prematurity and admission to neonatal intensive care unit, the rate of SGA was increased in offspring born to overweight/obese mothers who underwent bariatric surgery, as compared to offspring of mothers without operations (relative risk 1.93; 95% confidence interval 1.65–2.26) [78]. A shortage of maternal micronutrients resulting from bariatric surgery likely adds to the undesirable pregnancy outcomes [79], and thus, regular monitoring and nutritional recommendations are required for the pre-conception and gestational phases.

Furthermore, a recent systematic review suggested that weight reduction through lifestyle intervention in women with obesity prior to infertility treatment might cause an increase in the rate of miscarriages [33]. In the Norwegian Fit for Delivery study, the risk for SGA increased with higher pre-pregnancy (p-trend = 0.036) or early-pregnancy (p-trend = 0.043) diet quality scores [80]. The authors suggested that behaviors focused on energy balance might lead to a shortage of fuel for child development [80]. However, the relationship was no longer significant in a model adjusted for other maternal variables that might explain the higher SGA rate associated with the diet scores. In a French cohort study [81], a restrictive diet and weight loss prior to pregnancy resulted in increased gestational weight gain, which is a known risk factor for large-for-gestational-age birth weight and overweight in children [82]. Another study showed an increased risk for SGA in normal-weight women after weight loss prior to pregnancy (odds ratio 1.76; 95% confidence interval 1.10–2.81), while there were no effects on birth weight in children born to women with overweight/obesity (BMI 25–40 kg/m^2^) who had pre-pregnancy weight loss, although the sample size of this subgroup was small [83].

These findings highlight the challenge for a public health campaign targeting women who plan a pregnancy. While supervised weight loss prior to pregnancy might be beneficial to women who are overweight or obese, overly health-conscious women with normal weight might also follow the guidance addressed at women with higher BMI and lose weight or restrict their eating habits at the risk of nutritional deficiencies and SGA birth weight of babies. The timing, method, and extent of desirable weight loss in women with overweight or obesity have to be assessed in future studies.

Following the weight loss of men through bariatric surgery, different effects on sperm quality at 1–2 years after surgery are reported. In some case studies, reduced sperm quality or increased rates of infertility were reported [84–86], whereas others found improvements [87] or no changes of sperm quality [88]. It remains to be determined if the timing of weight loss in fathers prior to conception is associated with later health outcomes in the offspring. In male mice, pre-pregnancy undernutrition negatively affected sperm quality and led to adiposity and dyslipidemia in their offspring later in life [89]. Supplementation of antioxidants and vitamins of the paternal diet prior to mating them restored offspring metabolic health [89]. If dietary supplementation in overweight/obese men is beneficial for offspring health, remains to be studied.

These results suggest that weight loss may lead to metabolic disturbances affecting gamete quality and if in close proximity to conception might increase the risk for metabolic diseases in the offspring. Therefore, caution is advised when couples planning to become pregnant lose weight prior to conception. High-quality intervention trials are needed that test the timing and extent of advisable weight loss before conception to harvest potential beneficial effects for mother and child.

## Improving diet and health rather than focusing on weight loss prior to conception

While motivation to change one’s lifestyle might be increased in persons planning to have a child, the obstacles to achieve weight loss are no less. In addition, advanced age in mothers [90] and fathers [91] is another risk factor for adverse pregnancy and child outcomes. Therefore, the risk stemming from increased BMI has to be weighed against the risk from advanced age, as weight loss and weight maintenance might take considerable time. One study even found low willingness in women in their mid-thirties (mean age 34.5 years) with overweight or obesity to postpone fertility treatment in order to actively reduce weight [92]. These findings should be considered in pre-conceptional health advice.

If weight loss is not possible due to advanced age or lack of means or willingness, then smaller changes in the dietary patterns might be an option. To shift the focus from weight reduction to an improved diet might also improve adherence to the advice, as persons with overweight or obesity and a history of weight loss attempts are less likely to lose weight in subsequent interventions [93]. Overweight and obesity seem to be associated with lesser diet quality in pregnant women [94, 95]. Women who adhered to a Mediterranean style dietary pattern seem to have higher pregnancy rates compared with women eating a Western style diet [96]. However, in an analysis of the Nurses’ Health Study II, peri-pregnancy diet quality was not associated with overweight or obesity in offspring at 12–23 years of age, when adjusted for maternal BMI [97].

Diet composition also affects sperm function and quality [98, 99]. In obese mice, a diet and/or exercise regime helped to restore sperm parameters, even if obesity was sustained [100]. However, epigenetic variation in sperm from mice provided with different diets (control diet, low-protein diet, and HFD) was not dependent on the diet type, and the authors concluded that there is no immediate effect of diet on the metabolism of the subsequent generation [101]. These three diets differed in their macronutrient compounds, but diet quality is not only measured in fat, protein, and carbohydrates. Various plant compounds including phytochemicals affect epigenetic mechanisms and might influence epigenetic programming in the embryo as reviewed elsewhere [102]. In addition, inadequate micronutrient intake, which is common in energy-dense Western dietary patterns [103] and in pre-conception overweight and obesity [104], might affect pregnancy and child outcomes [105], due to different potential mechanisms. First, the micronutrient density of a diet appears to impact hunger perception, and a micronutrient-dense diet has been shown to attenuate the negative experience of hunger [106]. Vice versa, deficiencies of micronutrients could potentially exacerbate hunger and thereby drive excessive calorie consumption and subsequent weight gain. Adequate intake is therefore a prerequisite for weight maintenance, especially in the peri-conception phase. Second, low concentrations of micronutrients, especially those acting as methyl donors, increased the cellular expression of genes that regulate adipogenesis and lipogenesis and altered epigenetic processes [107–109]. Even though micronutrient deficiencies early in human pregnancy have been shown to be associated with maternal obesity and dyslipidemia, the implications for offspring metabolic health are not fully understood [105, 110]. One study in humans showed that low maternal folate levels were associated with a slight increase in offspring BMI at age 5–6 years [111]. Besides, there seems to be a role for the father’s folate status at conception and offspring embryonic growth [112].

To date, the effects of pre-conception micronutrient deficiency on child outcomes including stunting are mostly studied in low- and middle-income countries in the context of undernourishment [113], while the consequences of energy-rich, micronutrient-poor diets at conception remain to be elucidated. Therefore, research is warranted that assesses the role of peri-conception dietary patterns rich in fruit and vegetables, micronutrients, and beneficial lipids on long-term health outcomes in offspring against a background of excessive energy intake as in overweight and obese mothers.

## Outlook

The focus of pre-conception intervention strategies needs to shift from women to couples, as emerging data show that fathers contribute to the health trajectory of their progeny. Future studies should also address possible interactions between maternal and paternal lifestyle and health status. While overweight and obesity in parents are risk factors for undesirable health outcomes in their offspring, weight loss in close proximity to a planned pregnancy should not be the main focus of interventions. An improvement of diet quality might be an alternative to prevent or lessen obesity-associated adverse health outcomes in offspring. However, if and what kind of change in parental pre-conception diets could protect the offspring from undesirable metabolic programming warrants future studies. Studies that aim to answer these questions are highly ambitious due to the challenges in recruitment, the number of subjects needed, the length of the follow-up, and the costs; however, they are needed.

The peri-conceptional period sets the stage for a healthy life of a person. However, how a life is lived, is determined by its environment. While the pre-conceptional phase might be a time for motivation to improve one’s health, pre-conceptional health is also a societal issue and should be addressed on a global, national, and community scale. Healthy choices should be attainable to everybody to improve public health in general. Following a life-course approach will improve health for couples before, during, and after pregnancy and improve the health of the future generations.
